# Twin hearts—minimally invasive mitral valve repair in twin sisters with mitral annular disjunction and Loeys-Dietz syndrome: a case series

**DOI:** 10.1093/jscr/rjad344

**Published:** 2023-06-17

**Authors:** Stelios Ioannou, George Shiakos, Theodoros Ntoskas, Elias Papasavvas, Violetta Anastasiadi, Nikoleta Betsimea Loizides, Petros Mavrommatis, Ioannis Tzanavaros

**Affiliations:** Cardiac Innovation Center of Apollonion Private Hospital, Nicosia, Cyprus; Cardiac Innovation Center of Apollonion Private Hospital, Nicosia, Cyprus; Mediterranean Hospital of Cyprus, Limassol, Cyprus; Apollonion Private Hospital, Nicosia, Cyprus; Karaiskakio Foundation, Nicosia, Cyprus; Saint Andrews Cardiological Centre Paphos, Paphos, Cyprus; Cardiac Care Centre, Paphos, Cyprus; Cardiac Innovation Center of Apollonion Private Hospital, Nicosia, Cyprus

## Abstract

In this case report, we present 31-year-old twin sisters diagnosed with severe Barlow mitral valve prolapse, mitral annular disjunction and presence of lateral mid-wall fibrosis diagnosed on MRI as well as ventricular arrhythmias, and a very rare variant of Loeys-Dietz syndrome, being referred to our center for surgical repair. Genetic testing detected pathogenic variants of clinical significance in SMAD3 and KCNH2 genes that are associated with autosomal dominant disease of Loeys-Dietz syndrome. Due to the presence of severe mitral valve regurgitation, the first patient was referred for minimally invasive mitral valve repair that was performed successfully. Before discharge, a subcutaneous ICD implantation was performed as primary prevention against malignant ventricular arrhythmias and sudden cardiac death. Her twin sister presented with the identical diagnosis and underwent the same surgical procedure with S-ICD implantation a few months later.

## INTRODUCTION

We share a case report of twin sisters genetically diagnosed with a rare variant of Loeys-Dietz syndrome, presenting also with a severe Barlow mitral valve prolapse and mitral annular disjunction (MAD). Sudden cardiac death of a younger sister at the age of thirteen is reported in the family history.

Mitral annular disjunction is a structural abnormality of the mitral annulus fibrosus, which has been described by pathologists to be associated with mitral leaflet prolapse. Mitral annular disjunction is a common finding in patients with myxomatous mitral valve diseases. The prevalence of mitral annular disjunction should be checked routinely during presurgical imaging. Otherwise, mitral annular disjunction itself might be an arrhythmogenic entity, irrespective of the presence of mitral valve prolapse (MVP) that can lead to sudden cardiac death [1].

Its recognition in transthoracic and confirmation on transesophageal echocardiography is important to facilitate optimal mitral valve repair. The modification of the repair technique allows surgical correction of the annular disjunction, which seems to optimize long-term results in these challenging cases [2]. The importance of genetically diagnosed variations of Loeys-Dietz syndrome should be highlighted, as in rare cases, the combination of mitral annular disjunction can be of clinical significance or even fatal if not treated.

Mutations in the SMAD3 gene have been found to cause Loeys-Dietz syndrome type III. The SMAD3 gene provides instructions for making the protein necessary for the signaling pathway of the transforming growth factor-beta (TGF-β). Through the TGF-β signaling pathway, the SMAD3 protein also influences many aspects of cellular processes, including cell growth and division (proliferation), cell movement (migration) and controlled cell death (apoptosis) [3]. The overactive signaling pathway leads to dysregulated cell proliferation and gene activation. These changes lead to the abnormalities typical of Loeys-Dietz syndrome type III.

On the other hand, the KCNH2 gene belongs to a family of genes that provide instructions for making potassium channels. Those channels are found in the cardiac muscles and are responsible for the generation and transmission of electrical signals. Health conditions related to genetic changes of this gene are Romano-Ward syndrome, Short-QT syndrome, Familial atrial fibrillation and can be even associated with acquired long QT syndrome and increased risk for sudden cardiac death [4].

## CASE SERIES

In this case report, we present 31-year-old twin sisters diagnosed with severe Barlow mitral valve prolapse and mitral annular disjunction and presence of lateral mid-wall fibrosis diagnosed on MRI as well as ventricular arrhythmias, and a very rare variant of Loeys-Dietz syndrome type III, being referred to our center for surgical repair. The sudden death of their otherwise healthy thirteen-year-old sister a few years ago due to unknown causes prompted further cardiological evaluation.

**Figure 1:**
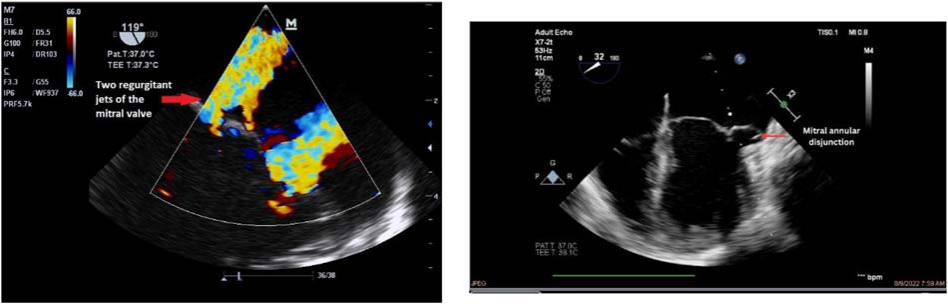
Preoperative echocardiography of patient 1.

**Figure 2:**
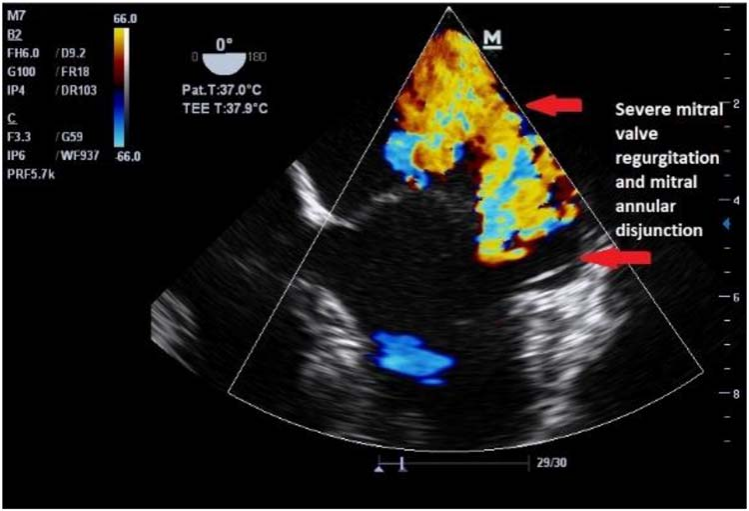
Preoperative echocardiography of patient 2.

**Figure 3:**
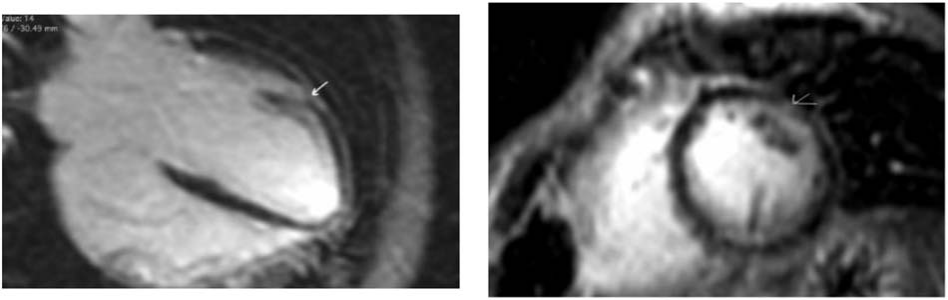
Midwall fibrosis in the mid-anterolateral wall of patient 1.

**Figure 4:**
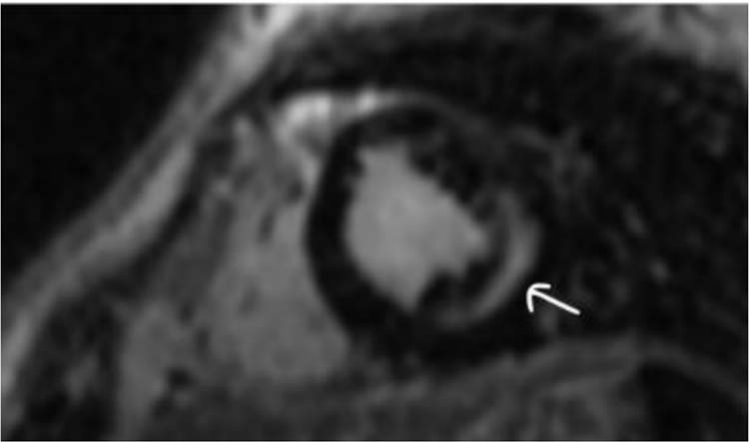
Midwall fibrosis in the mid-anterolateral wall of patient 2.

**Figure 5:**
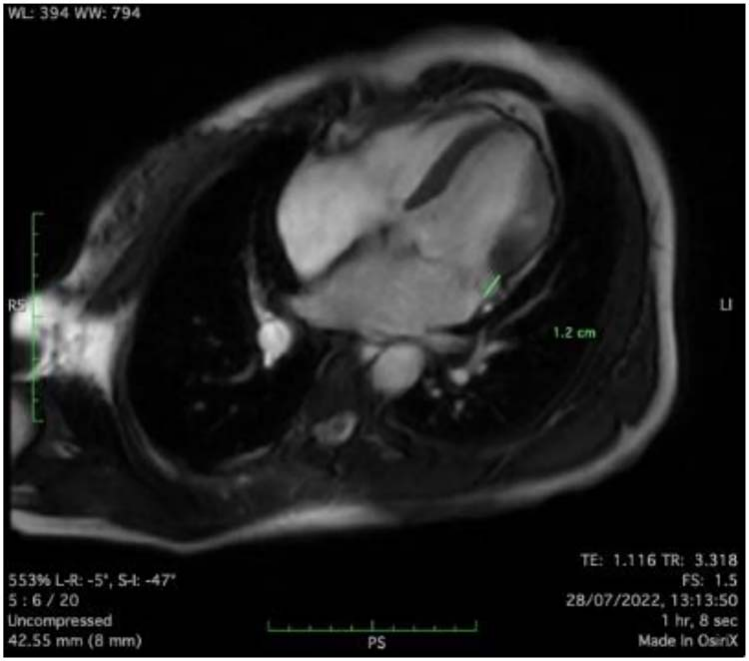
Mitral annular disjunction 12 mm of patient 1.

**Figure 6:**
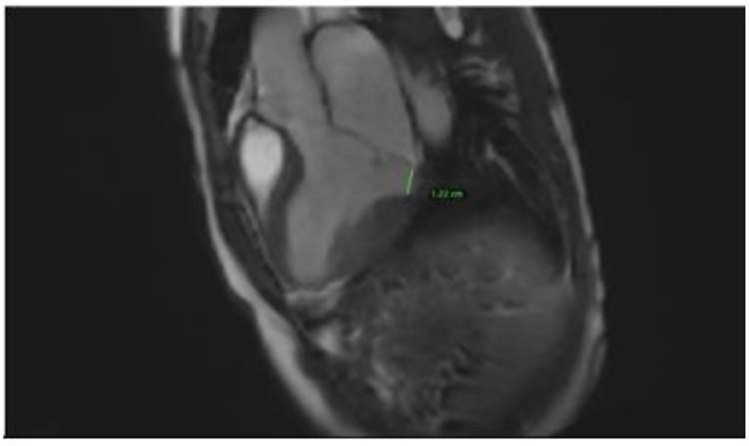
Mitral annular disjunction 12 mm of patient 2.

Clinically, both patients complained of worsening palpitations over the last few months with no associated dyspnea or tiredness. A Holter examination recorded 14 444 VES and 212 NSVT, with very similar results in both sisters. The echocardiography revealed a myxomatous Barlow mitral valve with anterior and posterior leaflet prolapse and two regurgitant jets resulting in moderate to severe mitral valve regurgitation as well as mitral annular disjunction in both cases (Figs 1 and 2). A cardiac MRI on a 3 T Lumina Siemens revealed at the mid-left ventricular level, a midwall fibrosis in the midlateral myocardium (Figs 3 and 4) and confirmed a mitral annular disjunction of 12 mm (Figs 5 and 6). Additional findings included a dilated LV with mildly depressed contractility, no visible myocardial scarring, no structural heart disease and a normal sized RV with mildly depressed contractility.

Taking into consideration the presence of moderate to severe mitral valve regurgitation and the arrhythmogenic potential of the diagnosed mitral annular disjunction especially with a reported case of sudden cardiac death in the family history, surgical repair in both sisters was planned. Both patients were instructed to avoid strenuous physical activities, and both were placed on low-dose beta-blocker therapy preoperatively.

**Figure 7:**
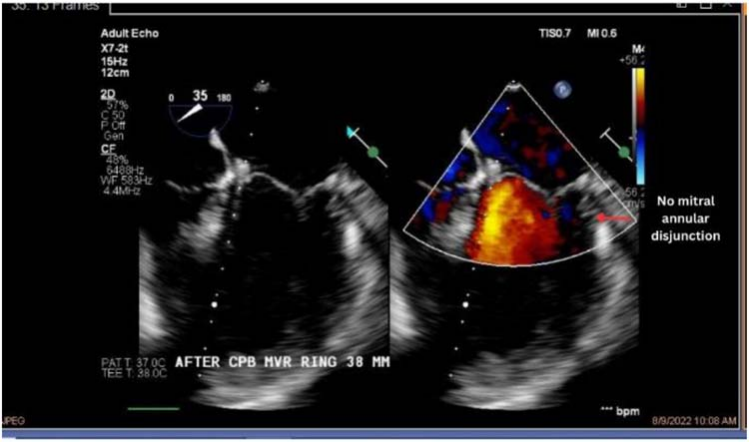
Postoperative echocardiography of patient 1.

**Figure 8:**
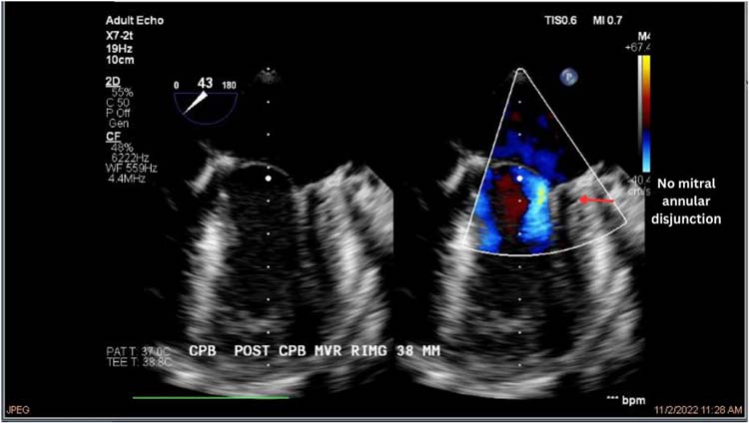
Postoperative echocardiography of patient 2.

Both sisters underwent a minimally invasive procedure through a right-sided lateral minithoracotomy performed at the level of the third or fourth intercostal spaces. Cardiopulmonary bypass was established through a femoral access and cardioplegic cardiac arrest was achieved through a modified Del Nido solution (20 ml/kg). Mitral valve repair was performed through isolated annuloplasty using in both cases a 38-mm semi-rigid annuloplasty ring. Transesophageal echocardiography after repair revealed excellent surgical results, with no rest regurgitation, no SAM of the mitral valve and most importantly no detectable mitral annular disjunction (Figs 7 and 8). In both cases, being performed several months apart, the patients were extubated in the operating room and transferred to our ICU for further observation.

The postoperative course was largely uneventful and both sisters made an excellent recovery. Both were kept on monitoring throughout the in-hospital stay and no relevant ventricular or supraventricular arrhythmias were documented under the established beta-blocker therapy. After individual risk stratification and taking in to account the reported case of sudden cardiac death within the family, and the recorded episodes of NSVT preoperatively, we proceeded with an S-ICD implantation in both cases before discharge from the hospital. Follow up at 7 and 30 days after discharge confirm excellent operative results and both patients remain asymptomatic. Analysis of the S-ICD data in a postoperative check, revealed no episodes of ventricular tachycardia in none of the patients.

## DISCUSSION

Rare cases like this lead us to consider the importance of genetic examinations of patients as well as their families with heart diseases and elastopathies. On the other hand, mitral annular disjunction itself can be an arrhythmogenic entity that can lead to sudden cardiac death. This rare condition is seen in about 30% of the patients with mitral valve prolapse based upon different populations and imaging modalities [5]. As its diagnosis may have further implications in the planning of the surgical therapy as well as postoperative rhythmological considerations, mitral annular disjunction must be considered in all patients with mitral valve prolapse especially in the setting of Barlow’s disease.

In our reported case, both patients underwent an S-ICD implantation before discharge as they were both considered to have a high risk of sudden cardiac death. The role of ICD implantation as primary prevention in such cases is unclear as available data are limited. We consider an individual risk stratification of such patients appropriate taking into consideration the arrhythmogenic potential of MAD, myocardial fibrosis, medical history of malignant arrhythmias and family history. In our case, both sisters presented with a large longitudinal distance of MAD in magnetic resonance, severe Barlow’s disease at a young age, and a reported case of sudden cardiac death in the family all of which are predictors of arrhythmias in MAD [6]. The choice of subcutaneous ICD implantation vs. transvenous was made solely on the patient’s age and preservation of their quality of life.

To conclude, in this report, we present a case of successful mitral valve repair in twin sisters with severe Barlow mitral valve prolapse, mitral annular disjunction and a very rare variant of Loeys-Dietz syndrome. Although, the hereditary nature of Barlow’s disease is well documented, this is to our knowledge the first reported case of twins with severe Barlow mitral valve prolapse, MAD and lateral mid-wall myocardial fibrosis being treated successfully in an identical manner. The significance of the diagnosis of MAD should be emphasized especially in younger patients with MVP and further research is required as to the role of ICD implantation as primary prevention for sudden cardiac death in such patients.
